# A Clinical Treatise on Diseases of the Liver

**Published:** 1879-04

**Authors:** 


A CLINICAL TREATISE ON DISEASES OF THE LIVER. By DR. FRIED. THEO. FRERICHS, Professor of Clin. Med. in the Univ of Berlin, etc., etc. In three volumes. Vol. 1—Translated by Chas. Murchison, M.D., F.R.C P.; Physician to the London Fever Hospital, etc., etc. New York: Wm. Wood & Co., Publishers. 1879.

This is one of the series of Messrs. Wood & Co.'s Library of Standard Medical Authors, and is the first of the three volumes that it is proposed to devote to this subject. It contains the history and physical diagnosis of diseases of the liver, with certain individual affections, as icterus, acholia, acute and chronic atrophy, fatty liver, pigment liver and hemorrhages and hyperæmias of the organ. The more important diseases will find a place in the second volume.

The author's clinical observations on certain obscure forms of jaundice are supplemented by explanatory theories of its origin and character that call in question and decidedly conflict with some former hypotheses. Why there is such a divergence in the course of the two products of the hepatic function, bile and sugar, is a question still inexplicable, but to the author the fact has a bearing in determining and forming a theory as to the origin of icterus in certain conditions. That the bile is preformed in the blood is positively denied, yet the view that jaundice results from the mechanical obstruction to the discharge of bile from the bile ducts, is admitted. Suppression, however, will not produce the disease, while in morbid conditions of the blood arising from the introduction of zymotic poisons into the circulation, the normal amount of bile acids that usually accompany the sugar in the hepatic vein, are changed into bile pigment instead of the usual metamorphosis into urinary pigment occurring.

Altogether the work seems well worthy a special niche in the library of every enterprising physician, as it treats exhaustively of a pathogenetic element with which the practitioner comes in daily contact, and which is frequently the source of no little annoyance.

